# High-dose vitamin-C induced prolonged factitious hyperglycemia in a peritoneal dialysis patient: a case report

**DOI:** 10.1186/s13256-021-02869-4

**Published:** 2021-05-21

**Authors:** Olivier Lachance, François Goyer, Neill K. J. Adhikari, Marie-Hélène Masse, Jean-François Bilodeau, François Lamontagne, Marc-André Leclair

**Affiliations:** 1 Department of Critical Care Medicine, Université de Montréal, 5400 Boul Gouin Ouest, Montreal, QC H4J 1C5 Canada; 2 Department of Medical Biochemistry, Université de Sherbrooke, 3001 12e Avenue N, Sherbrooke, QC J1H 5H3 Canada; 3 Department of Critical Care Medicine, Sunnybrook Health Sciences Centre and Interdepartmental Division of Critical Care Medicine, University of Toronto, 2075 Bayview Ave, Toronto, ON M4N 3M5 Canada; 4 Centre de recherche du Centre Hospitalier Universitaire de Sherbrooke, 3001 12e Avenue N, Sherbrooke, QC J1H 5H3 Canada; 5 Department of Medicine, Université de Sherbrooke, 3001 12e Avenue N, Sherbrooke, QC J1H 5H3 Canada

**Keywords:** Ascorbic acid, Vitamin C, Intensive care, Septic shock, Sepsis, Peritoneal dialysis

## Abstract

**Background:**

High-dose vitamin C is increasingly used for sepsis and more recently for coronavirus disease 2019 (COVID-19) infections. Proponents argue that the low cost and near perfect safety profile of vitamin C support its early adoption. Yet, adverse events might be underreported and underappreciated.

**Case presentation:**

We report a 73-year-old non-diabetic white man with end-stage renal disease on peritoneal dialysis admitted to the intensive care unit with septic shock that was suspected to be due to peritonitis. The patient was enrolled in LOVIT (Lessening Organ Dysfunction with VITamin C; ClinicalTrials.gov identifier: NCT03680274), a randomized placebo-controlled trial of high-dose intravenous vitamin C. He developed factitious hyperglycemia, as measured with a point-of-care glucometer, that persisted for 6 days after discontinuation of the study drug, confirmed to be vitamin C after unblinding. He also had short-lived iatrogenic coma because of hypoglycemia secondary to insulin administration. These events triggered a protocol amendment.

**Conclusions:**

Although factitious hyperglycemia has been reported before using certain glucometers in patients treated with high-dose vitamin C, the persistence of this phenomenon for 6 days after the discontinuation of the therapy is a distinguishing feature. This case highlights the importance of monitoring glucose with a core laboratory assay for up to a week in specific populations, such as patients on peritoneal dialysis.

## Background

High-dose vitamin C therapy for sepsis is being actively debated in the critical care community. A single-center before–after study suggested that high-dose intravenous vitamin C combined with thiamine and hydrocortisone dramatically decreases mortality, organ failure, and vasopressor requirements in septic shock [1], which was contradicted by a recent multicenter open-label randomized clinical trial [2]. Another trial found that high-dose intravenous vitamin C reduced 28-day mortality in patients with sepsis and acute respiratory distress syndrome [3].

Skeptics have criticized early reports of efficacy, while enthusiasts argue that the high burden of sepsis and low cost and remarkable safety of vitamin C justify early adoption. Yet, vitamin C therapy has already been associated with factitious hyperglycemia and harmful iatrogenic hypoglycemia [4, 5], causing death in at least one report [6]. LOVIT (Lessening Organ Dysfunction with VITamin C) is a multicenter randomized controlled trial (ClinicalTrials.gov identifier: NCT03680274) examining the effects of intravenous vitamin C (50 mg/kg every 6 hours for 96 hours) in septic patients in the intensive care unit (ICU). A patient in this trial had factitious hyperglycemia leading to insulin administration and hypoglycemic coma, which triggered a protocol amendment, highlighting the hazards of such therapy.

## Case presentation

A 73-year-old non-diabetic (HbA1c 5.7%) white man with end-stage renal disease on peritoneal dialysis was admitted with diarrhea, fever, and hypotension. At home, the patient was on automated nocturnal peritoneal dialysis (6 × 2 L × 2.5% over 8 hours) with 2 L of icodextrin as a daytime dwell. Physical examination was unremarkable except for abdominal distension. Sepsis due to peritonitis was suspected, and he received intravenous fluids, vasopressors, and broad-spectrum antibiotics. Blood cultures grew *Salmonella* spp. The patient continued to receive peritoneal dialysis, with three daytime exchanges of 2.5% or 4.25% dextrose and one night exchange of icodextrin. The patient had some urine output at baseline but became anuric following hospital admission.

The patient consented to participation in the LOVIT trial 18 hours after admission to the ICU. Before the first dose of study drug, he received subcutaneous insulin due to sepsis-associated hyperglycemia. Capillary blood glucose was monitored with the Accu-Chek Inform II glucometer (F. Hoffmann-La Roche Ltd.). On hospital day 5, he received intravenous insulin for worsening hyperglycemia, after which he became unconscious. A blood sample sent to the core laboratory and tested using a hexokinase assay showed severe hypoglycemia (1.7 mmol/L; normal > 4 mmol/L). Insulin was discontinued, 50% dextrose was administered, and his cognitive status normalized.

**Fig. 1:**
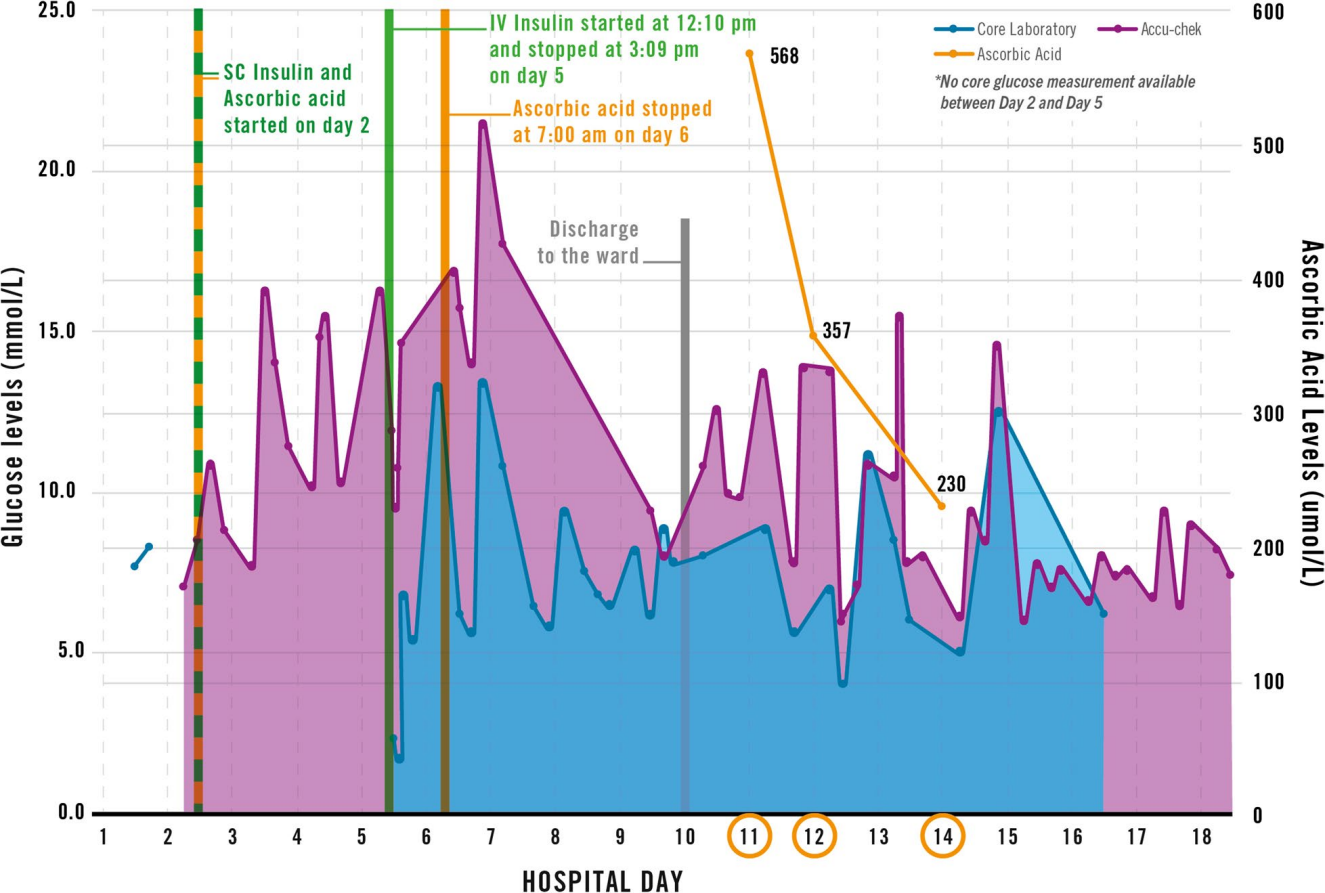
Glucose values from glucometer and core laboratory assay before, during, and after vitamin C administration. Note that core laboratory measurements were not available between day 2 and day 5

After unblinding, research staff confirmed that he had been receiving vitamin C. He was discharged to the ward on hospital day 10. An ascorbic acid level of 568 μmol/L (normal range: 30–114 μmol/L) was measured using a spectrophotometric dinitrophenylhydrazine assay 5 days after discontinuing vitamin C therapy, on hospital day 11, and important differences between blood glucose measured by the core laboratory and point-of-care glucometers persisted until hospital day 12 (Fig. 1). Following this event, the LOVIT trial protocol was amended to mitigate the risk of factitious hyperglycemia. The patient was discharged home without any apparent sustained harm.

## Discussion

High-dose vitamin C treatments is increasingly used outside of trials for various conditions such as sepsis and more recently for coronavirus disease 2019 (COVID-19) infections [7]. This case highlights that the adverse effects of high-dose vitamin C treatment may not be fully appreciated and that claims of vitamin C’s absolute safety are likely unfounded. Although factitious hyperglycemia on point-of-care glucometers in the presence of elevated plasma ascorbic acid is documented [4, 8], the persistence of factitious hyperglycemia for 6 days after discontinuing vitamin C therapy is a distinguishing feature of this case. An earlier study in patients with burn injury suggested that measuring glucose with a core laboratory method for 24 hours after discontinuation of high-dose vitamin C was a safe approach [4]. The severity and the persistence of this episode suggests that a longer period of monitoring of blood glucose, up to 1 week, might be necessary, perhaps particularly in patients with reduced renal function and those receiving peritoneal dialysis. During this extended period, glucose should be monitored with a core laboratory assay, or other device whose glucose levels are documented to be reliable in the presence of high plasma ascorbic acid concentrations, until no discrepancy with point-of-care glucometers can be demonstrated.

In people with normal kidney function, ascorbic acid is mostly excreted renally [9]. Peritoneal dialysis may have contributed to this patient’s course, since pharmacokinetic studies have shown that peritoneal dialysis does not reliably clear plasma oxalic acid, which is a major end product of ascorbic acid oxidation [10]. In addition, icodextrin has been associated with factitious hyperglycemia due to an accumulation of its metabolites, notably maltose [11], although modern glucometers (including the Accu-Chek Inform II) are reportedly impervious to this interference. However, *in vitro* studies have confirmed significant positive interference on the Accu-Chek Inform II for high plasma concentrations of vitamin C (850–1700 μmol/L) [8]. The mechanism is related to the electrochemical method of measuring glucose and oxidation of vitamin C at the electrode surface, which creates a current yielding a false signal of hyperglycemia. While some evidence suggests that certain glucometers (for example, StatStrip [Nova Biomedical]) are more reliable than others in the context of high-dose vitamin C therapy, clinical experience with such devices remains extremely limited [12]. Since glucometers are ubiquitous on medical wards, the cost of acquiring, calibrating, and regularly testing point-of-care devices impervious to ascorbic acid interference will need to be considered as an additional cost of high-dose vitamin C treatment, which may not be justified unless a clear benefit of this treatment can be demonstrated.

This event reinforces the importance of rigorously evaluating novel interventions, even if they are relatively inexpensive and routinely available. The dose of vitamin C required to improve outcomes in sepsis may exceed that required to normalize plasma levels [13], since scavenging of oxygen radicals and other mechanisms of action may require high intracellular ascorbic acid concentrations. Adverse events are underappreciated and underreported. To mitigate these effects, research should address vitamin C’s optimal dosing and pharmacokinetics in critically ill septic patients, including those with renal failure.

## Conclusion

Before the widespread adoption of high-dose vitamin C for the treatment of septic shock, additional randomized trials should assess its safety and potential side effects, which might otherwise be underreported. In certain populations, such as patients undergoing peritoneal dialysis, the risk of persistent factitious hyperglycemia while using point-of-care glucometers is of particular importance. In this population, measurement of blood glucose should be done with core laboratory assay for up to a week after vitamin C discontinuation.

## Data Availability

All data generated or analyzed during this study are included in this published article.

## Abbreviation

LOVIT: Lessening Organ Dysfunction with VITamin C
